# Combined free-running four-dimensional anatomical and flow magnetic resonance imaging with native contrast using Synchronization of Neighboring Acquisitions by Physiological Signals

**DOI:** 10.1016/j.jocmr.2024.101006

**Published:** 2024-02-02

**Authors:** Mariana B.L. Falcão, Adèle L.C. Mackowiak, Giulia M.C. Rossi, Milan Prša, Estelle Tenisch, Simone Rumac, Mario Bacher, Tobias Rutz, Ruud B. van Heeswijk, Peter Speier, Michael Markl, Jessica A.M. Bastiaansen, Matthias Stuber, Christopher W. Roy

**Affiliations:** a Department of Diagnostic and Interventional Radiology, Lausanne University Hospital (CHUV) and University of Lausanne (UNIL), Lausanne, Switzerland; b Department of Diagnostic, Interventional and Pediatric Radiology (DIPR), Inselspital, Bern University Hospital, University of Bern, Bern, Switzerland; c Translation Imaging Center (TIC), Swiss Institute for Translational and Entrepreneurial Medicine, Bern, Switzerland; d Woman, Mother, Child Department, Lausanne University Hospital and University of Lausanne, Lausanne, Switzerland; e Siemens Healthcare GmbH, Erlangen, Germany; f Service of Cardiology, Centre de Resonance Magnétique Cardiaque (CRMC), Lausanne University Hospital and University of Lausanne, Lausanne, Switzerland; g Department of Radiology, Feinberg School of Medicine, Northwestern University, Chicago, Illinois, USA; h Department of Biomedical Engineering, Northwestern University, Chicago, Illinois, USA; i Center for Biomedical Imaging (CIBM), Lausanne, Switzerland

**Keywords:** 4D flow, SyNAPS, FISS, Free-running, Anatomy and flow MRI, Dynamic vessel segmentation

## Abstract

**Background:**

Four-dimensional (4D) flow magnetic resonance imaging (MRI) often relies on the injection of gadolinium- or iron-oxide-based contrast agents to improve vessel delineation. In this work, a novel technique is developed to acquire and reconstruct 4D flow data with excellent dynamic visualization of blood vessels but without the need for contrast injection. Synchronization of Neighboring Acquisitions by Physiological Signals (SyNAPS) uses pilot tone (PT) navigation to retrospectively synchronize the reconstruction of two free-running three-dimensional radial acquisitions, to create co-registered anatomy and flow images.

**Methods:**

Thirteen volunteers and two Marfan syndrome patients were scanned without contrast agent using one free-running fast interrupted steady-state (FISS) sequence and one free-running phase-contrast MRI (PC-MRI) sequence. PT signals spanning the two sequences were recorded for retrospective respiratory motion correction and cardiac binning. The magnitude and phase images reconstructed, respectively, from FISS and PC-MRI, were synchronized to create SyNAPS 4D flow datasets. Conventional two-dimensional (2D) flow data were acquired for reference in ascending (AAo) and descending aorta (DAo). The blood-to-myocardium contrast ratio, dynamic vessel area, net volume, and peak flow were used to compare SyNAPS 4D flow with Native 4D flow (without FISS information) and 2D flow. A score of 0–4 was given to each dataset by two blinded experts regarding the feasibility of performing vessel delineation.

**Results:**

Blood-to-myocardium contrast ratio for SyNAPS 4D flow magnitude images (1.5 ± 0.3) was significantly higher than for Native 4D flow (0.7 ± 0.1, p < 0.01) and was comparable to 2D flow (2.3 ± 0.9, p = 0.02). Image quality scores of SyNAPS 4D flow from the experts (M.P.: 1.9 ± 0.3, E.T.: 2.5 ± 0.5) were overall significantly higher than the scores from Native 4D flow (M.P.: 1.6 ± 0.6, p = 0.03, E.T.: 0.8 ± 0.4, p < 0.01) but still significantly lower than the scores from the reference 2D flow datasets (M.P.: 2.8 ± 0.4, p < 0.01, E.T.: 3.5 ± 0.7, p < 0.01). The Pearson correlation coefficient between the dynamic vessel area measured on SyNAPS 4D flow and that from 2D flow was 0.69 ± 0.24 for the AAo and 0.83 ± 0.10 for the DAo, whereas the Pearson correlation between Native 4D flow and 2D flow measurements was 0.12 ± 0.48 for the AAo and 0.08 ± 0.39 for the DAo. Linear correlations between SyNAPS 4D flow and 2D flow measurements of net volume (r^2^ = 0.83) and peak flow (r^2^ = 0.87) were larger than the correlations between Native 4D flow and 2D flow measurements of net volume (r^2^ = 0.79) and peak flow (r^2^ = 0.76).

**Conclusion:**

The feasibility and utility of SyNAPS were demonstrated for joint whole-heart anatomical and flow MRI without requiring electrocardiography gating, respiratory navigators, or contrast agents. Using SyNAPS, a high-contrast anatomical imaging sequence can be used to improve 4D flow measurements that often suffer from poor delineation of vessel boundaries in the absence of contrast agents.

## Graphical Abstract

**Figure:**
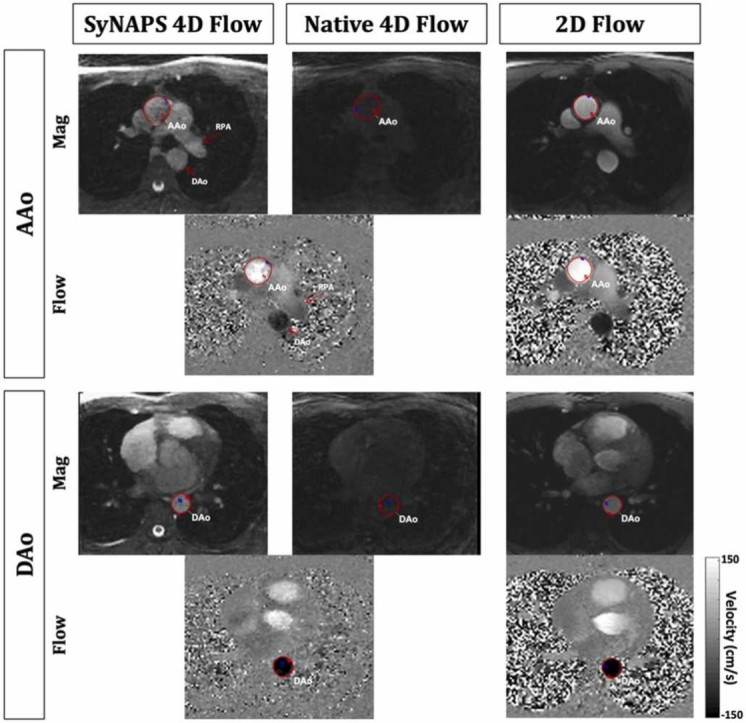

## 1 Background

Four-dimensional (4D) flow magnetic resonance imaging (MRI) is increasingly used in the diagnosis and management of cardiovascular diseases [1]. It provides a quantitative evaluation of blood flow in the heart and vessels with three-dimensional (3D) volumetric coverage. When compared to conventional two-dimensional (2D) flow MRI, simultaneous and retrospective interrogation of multiple vessels with 4D flow provides a significantly simplified scan protocol that is less user-dependent [2], [3]. Unfortunately, 4D flow MRI, which typically employs gradient recalled echo readouts, suffers from low contrast-to-noise when compared to 2D flow MRI, mainly due to the absence of in-flow signal enhancement. As a result, vessel delineation in 4D flow MRI is typically derived from a static phase-contrast magnetic resonance angiography reconstruction. This not only limits the dynamic segmentation of vessels and cardiac chambers for accurate flow assessment, but it also limits the overall visualization of cardiac structures (such as valves, vessel deformations, etc.) that could aid in clinical assessment while measuring flow. To improve signal-to-noise ratio and blood-to-myocardium contrast, 4D flow sequences are often acquired after injection of gadolinium- or iron-oxide-based contrast agents [1]. Nevertheless, there is a growing effort to limit the use of contrast agents due to their high cost and reported side effects [4], [5]. Therefore, the use of native contrast in 4D flow MRI is a preferred yet challenging solution.

Previous studies have explored the possibility of leveraging a separately acquired 3D anatomical scan to inform vessel and ventricular segmentation in a Cartesian 4D flow acquisition to more accurately measure blood flow [6], [7]. These studies, however, used prospective electrocardiography (ECG)-gating to synchronize reconstructions to the underlying heart rate and interpolation to provide the same number of cardiac phases for each sequence [6], [7], and, to account for respiratory motion, they used multiple-averages [6] or prospective respiratory gating to limit data-acquisition to end-expiration [7]. Furthermore, one of these studies used a gadolinium-based contrast agent to enhance image quality [7].

With the development of self-gating, cardiac motion can be processed retrospectively to sort the data into a user-defined number of cardiac phases without the need for data interpolation [8], [9]. Additionally, respiratory signals can be measured over time and used to either resolve or correct respiratory motion in the acquisitions [10], [11]. Nevertheless, self-gating methods derive physiological signals from the data itself, and therefore are inherently dependent on the contrast of a given sequence, making it difficult to synchronize reconstructions from separate acquisitions. Recently, Pilot Tone (PT) navigation has been proposed [12], [13], [14], providing cardiac and respiratory signals that are decoupled from the imaging sequence, and therefore could potentially be processed in similar fashion to synchronize reconstruction from different sequences.

In this study, a novel technique named **Sy**nchronization of **N**eighboring **A**cquisitions by **P**hysiological **S**ignals **(SyNAPS)** is introduced, with the goal of enabling the acquisition and reconstruction of 4D flow data with good blood-to-myocardium contrast without the need for contrast injection, by both leveraging the acquisition of an additional 3D anatomical sequence and PT navigation to retrospectively extract cardiac and respiratory signals. Using SyNAPS, two free-running [15] 3D radial datasets are acquired consecutively and processed in synchrony. The first dataset provides high contrast dynamic anatomical images with inherent fat suppression and uses a method called fast interrupted steady-state (FISS) [16], [17] that was adapted for 3D radial imaging [18]. The second dataset provides phase-contrast images, also using 3D radial imaging (3D radial PC-MRI), for quantification of blood flow velocity across the cardiac cycle [19]. The free-running datasets are continuously acquired without the need for ECG-gating or respiratory navigators because PT navigation [13], [14] is used to retrospectively synchronize the two acquisitions to inform a co-registered, joint cardiac motion-resolved, and respiratory motion-corrected 4D image reconstruction of the data. The resulting reconstructions are then combined to create 4D flow datasets with improved blood-to-myocardium contrast (“SyNAPS 4D flow”), which were compared to 4D flow measurements obtained only from free-running 3D radial PC-MRI data (“Native 4D flow”), and to 2D flow reference standard measurements.

The feasibility of SyNAPS was explored in a cohort of 13 healthy subjects and in 2 congenital heart disease patients. We tested the hypotheses that synchronizing these two free-running datasets to create SyNAPS 4D flow images enhances the visualization of the cardiac anatomy and great vessels, enables a dynamic segmentation of vessels, and improves the accuracy of 4D flow measurements of net volume and peak flow relative to their 2D flow reference counterparts.

## 2 Methods

### 2.1 Synchronization of Neighboring Acquisitions by Physiological Signals (SyNAPS)

**Fig. 1:**
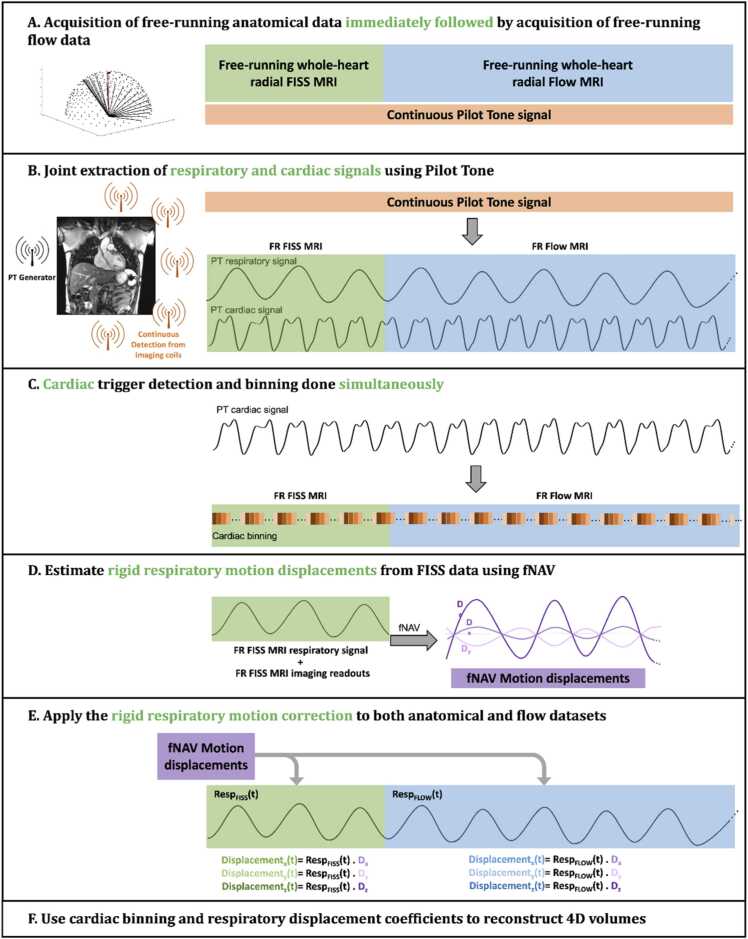
SyNAPS framework for synchronization of two free-running 3D radial MRI sequences. **A)** A free-running 3D radial fast interrupted steady-state sequence (FISS) [9], for a contrast free and natively fat suppressed whole-heart anatomical acquisition, and a free-running 3D radial PC-MRI sequence (flow) [10] are acquired sequentially. **B)** Cardiac and respiratory signals are extracted from the continuously acquired Pilot Tone (PT) signals [11]. **C)** From the extracted cardiac signals, triggers are selected to then perform cardiac binning of the data over time. **D)** From the extracted respiratory curves, displacement of the heart due to respiration is obtained from the FISS data using fNAV [16], [17], **E)** and then used to correct both sequences. **F)** The resulting cardiac binning and respiratory correction information is used to inform a k-t-sparse SENSE reconstruction adapted to each sequence to obtain 4D imaging volumes. The resulting magnitude images from 4D FISS and phase images from 4D flow are combined to create SyNAPS 4D flow. 3D: three-dimensional, 4D: four-dimensional, fNAV: focused navigation, FR: free-running, MRI: magnetic resonance imaging, PC-MRI: phase-contrast MRI, SyNAPS: Synchronization of Neighboring Acquisitions by Physiological Signals.

The proposed SyNAPS 4D flow framework consists of 6 steps as illustrated in Fig. 1. First, a pulse sequence with high blood-to-myocardium contrast (free-running 3D radial FISS [18]) and a pulse sequence with flow encoding (free-running 3D radial PC-MRI [19]) are acquired back-to-back with an acquisition interrupt of less than 2 s. To track cardiac and respiratory motion over the total acquisition time (∼14 min), a 12-channel body coil array with an integrated PT generator (Biomatrix Body 12, Siemens Healthcare, Erlangen, Germany) is used [13], [14], [20], [21], and the raw PT data are collected in parallel to each imaging sequence (Fig. 1A).

Second, cardiac and respiratory signals are estimated from the PT data based on a previously described framework [14], [22] for free-running 3D radial acquisitions. The PT signals that span the two acquisitions are continuously detected with the same receiver coils to facilitate extraction of cardiac triggers and respiratory curves as detailed below.

Third, as previously described [14], principal component analysis and independent component analysis were applied consecutively, first to reduce the computational complexity and then to accurately segregate the cardiac motion signal from the raw PT data. Following this step, cardiac triggers are extracted using adaptive filtering that targets frequencies within physiologically plausible ranges for cardiac motion. The local minima of the cardiac signal are used to define triggers that allow the binning of readouts from both acquisitions into the same number of cardiac phases (20 cardiac phases), with synchronization between sequences (Fig. 1C).

Fourth, respiratory curves are obtained using principal component analysis, used to reduce data complexity and to segregate the respiratory component [14], followed by adaptive low-pass filtering that targets frequencies within physiologically plausible ranges for respiratory motion [14] and detrending to reduce potential signal offsets between the two sequences or signal drift [15]. The bulk translational motion of the heart due to respiration is then corrected for both free-running FISS and free-running PC-MRI datasets using focused navigation (fNAV) as previously described [23], [24]. Briefly, the unitless respiratory curve derived from PT is multiplied by three initially unknown coefficients that describe the maximum displacement in millimeters of the heart over time along the three spatial dimensions. These coefficients are iteratively updated according to a metric for image blur (entropy of the gradient image) calculated over a region of interest containing the heart in the FISS images (Fig. 1D).

Fifth, the optimized displacement measurements are applied to the corresponding k-space data as a phase shift for both the FISS and PC-MRI acquisitions to correct for respiratory motion (Fig. 1E). Sixth, the data are binned into cardiac phases as described in the third step, and synchronized 4D FISS and 4D flow images are reconstructed separately using k-t-sparse sensitivity encoding (SENSE; Fig. 1F), which has previously been described for free-running FISS and free-running PC-MRI [15], [19], [25].

### 2.2 Study cohort

Thirteen healthy volunteers (9 female, 20–32 years old) and two patients with Marfan syndrome (2 female, 14–18 years old) were scanned on a 1.5T MAGNETOM Sola system (Siemens Healthcare, Erlangen, Germany) using a 12-channel body coil array with an integrated PT generator. All subjects provided written informed consent compliant with our institutional guidelines and approved by the local research ethics committee.

### 2.3 Image acquisition

As described in the previous section, free-running 3D radial FISS [18] and PC-MRI [19] sequences were acquired consecutively to obtain natively fat suppressed whole-heart anatomical images as well as whole-heart images with velocity encoding, respectively. The FISS [18] and PC-MRI [19] sequences used in this study were derived from previous published protocols.

For the free-running FISS sequence, a total of 2000 radial interleaves were acquired, each with 24 readouts (6 FISS modules and 4 readouts per FISS module) [17], [18]. Additional relevant parameters include repetition time = 2.94 ms, TE = 1.5 ms and total scan time = 3:06 min. For the free-running 3D radial PC-MRI sequence, 4820 interleaves were acquired, where readouts were repeated 4 times for balanced 4-point velocity encoding [14], [19]. A total of 21 radial readouts was acquired per interleave (1 superior inferior projection (SI) + (5 readouts × 4-point velocity encoding)). Additional relevant parameters include repetition time = 5.3 ms, echo time = 3.5 ms, venc = 150 cm/s, and total scan time = 8:59 min. Both free-running FISS and PC-MRI were acquired with the same field of view of (220 mm)^3^ (the effective field-of-view is doubled due to oversampling), spatial resolution (2.0 mm)^3^, and identical acquisition volume. The total scan time for each SyNAPS data acquisition was 12:05 min.

In addition to the free-running sequences, two standard breath-held 2D flow datasets at end-expiration were acquired for comparison. For each 2D flow dataset, one 2D plane was manually placed around two portions of the aorta, one was located near the base of the ascending aorta (AAo), and one was located at the descending aorta (DAo), between the third plane and the diaphragm. Sequence parameters for the 2D flow acquisitions were as follows: repetition time = 5.1 ms, TE = 2.9 ms, venc = 150 cm/s, field of view = 380 × 260 mm^2^, spatial resolution = 2.0 × 2.0 × 6.0 mm^3^, scan time = 0:15 min.

### 2.4 Free-running image reconstruction

All data were reconstructed on a workstation equipped with 2 Intel Xeon CPUs (Intel, Santa Clara, California, USA), 512 GB of RAM, and a NVIDIA Tesla GPU (Nvidia, Santa Clara, California, USA). For the k-t-sparse SENSE reconstruction [15], [19], [25], after normalizing each acquisition to the maximum signal from a gridded image reconstruction, regularization parameters for reconstructing free-running FISS datasets were 0.03 for total variation applied along the cardiac dimension and 0.015 for total variation applied along the spatial dimension, while the regularization parameters for free-running PC-MRI were 0.0075 in the cardiac dimension and 0.015 in the spatial dimension [24]. The free-running FISS reconstructions resulted in 4D FISS datasets (x-y-z-cardiac); reconstructions of free-running PC-MRI data returned 4D flow datasets (x-y-z-cardiac-velocity encode). The free-running FISS reconstruction took on average 39.3 ± 11.2 min, while the free-running PC-MRI datasets took around 3.5 ± 2.9 h to reconstruct. The variability depends on the total number of active coil elements and available computer resources during each reconstruction.

### 2.5 Data analysis

Post-reconstruction, each dataset contains magnitude (anatomy) and phase (velocity) images. In order to create the SyNAPS 4D flow datasets, the magnitude images from the 4D FISS datasets were extracted and then combined with the extracted velocity images from the 4D flow datasets. For comparison, Native 4D flow datasets (using both the magnitude and phase images of the free-running PC-MRI data) were also reconstructed. In both SyNAPS and Native 4D flow datasets, vessel planes were manually selected to match the same location as the 2D flow acquisitions in the AAo, DAo, and were analyzed using the Circle cvi42 software (Calgary, Alberta, Canada). After vessel segmentation, peak systole was detected for each of the three flow datasets to synchronize them for the remainder of the analysis. To quantify blood-to-myocardium contrast, two regions of interest were manually drawn, one in the DAo, and one in the myocardium, and the ratio of the signal intensity between these two regions was calculated.

The magnitude and phase images obtained for all flow datasets (2D flow, Native 4D flow, and SyNAPS 4D flow) and for all healthy volunteers and patients were placed in random order. Two blind readers (M.P., pediatric cardiologist, and E.T., radiologist) with 11 and 9 years of experience in CMR, respectively, were asked to score each magnitude-velocity pair shown in randomized order. Each observer provided one score of 0–4 regarding the feasibility of performing vessel delineation (0: No visibility for vessel delineation, 1: Limited visibility for vessel delineation, 2: Moderate visibility for vessel delineation, 3: Mild visibility for vessel delineation, 4: Excellent delineation of vessels) [26], followed by an additional binary score to establish if the magnitude image provided added value in the vessel delineation score (0: no added value by magnitude image, 1: added value provided by magnitude image). Statistical significance was measured using a Wilcoxon signed rank test with the Bonferroni correction for multiple comparisons.

Dynamic vessel tracing was performed on magnitude and phase images and was semi-automatically adjusted resulting in vessel area measurements for each time point in the cardiac cycle, for both the AAo and DAo, and for each of the SyNAPS 4D flow, Native 4D flow, and 2D flow datasets. For each subject (healthy volunteers and patients combined), the error in vessel area between SyNAPS 4D flow and 2D flow datasets was defined as the absolute difference between the two vessel area measurements averaged over time. In addition, the error in vessel area was similarly estimated between Native 4D flow and 2D flow datasets. The mean and standard deviation of the error in vessel area were reported for all subjects. Additionally, the Dice similarity coefficient was calculated by comparing vessels segmentations across all cardiac phases from SyNAPS 4D flow datasets to those from 2D flow datasets and, also by comparing those from Native 4D flow datasets to those from 2D flow datasets.

The flow rate curve was obtained throughout the cardiac cycle for each segmentation and the net volume and peak flow measurements were calculated. Differences in net volume and peak flow were estimated between SyNAPS 4D flow and 2D flow measurements as well as between Native 4D flow and 2D flow measurements.

Linear regression and Bland-Altman analysis were used to compare SyNAPS 4D flow, Native 4D flow, and 2D flow measurements. A Wilcoxon signed rank test was performed to assess the significant value of the differences measured.

## 3 Results

**Fig. 2:**
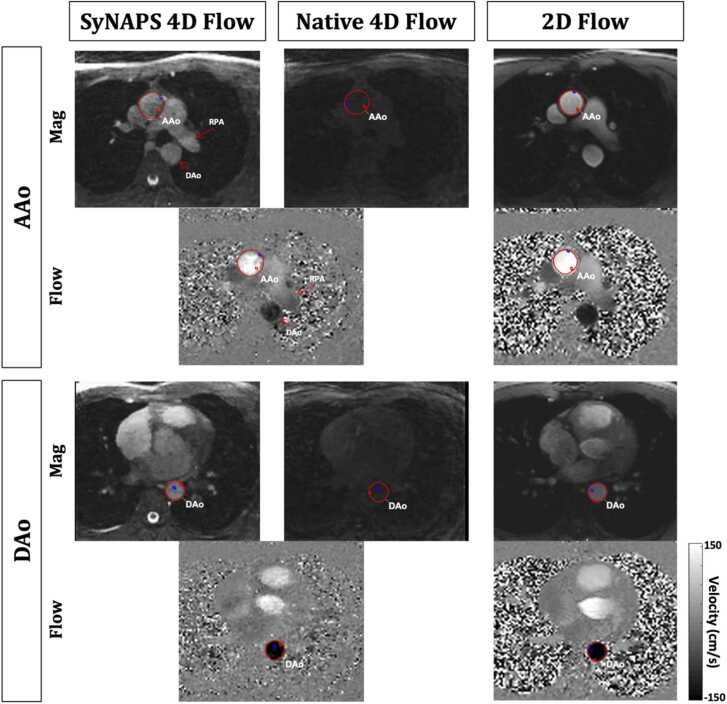
Comparison of SyNAPS 4D flow to Native 4D flow and to the reference 2D flow in one representative healthy volunteer. The anatomical images integrated in SyNAPS 4D flow display increased contrast when compared to Native 4D flow MRI. Additionally, heart and vessel structures are better depicted in SyNAPS 4D flow images, with similar contrast to that of 2D flow MRI. Images and vessel segmentations were captured from the image processing software, and blue and red dots inside the vessel segmentation represent maximum and minimum velocity voxels. 2D: two-dimensional, 4D: four-dimensional, AAo: base of the ascending aorta; DAo: the mid descending aorta; MRI: magnetic resonance imaging, RPA: right pulmonary artery, SyNAPS: Synchronization of Neighboring Acquisitions by Physiological Signals.

SyNAPS 4D flow images (Fig. 2) demonstrated a significant increase in blood-to-myocardium contrast (1.5 ± 0.3) when compared to their Native 4D flow counterpart (0.7 ± 0.1, p < 0.01). However, the blood-to-myocardium contrast from SyNAPS 4D flow images was lower than that found in 2D flow images (2.3 ± 0.9, p = 0.02).

**Fig. 3:**
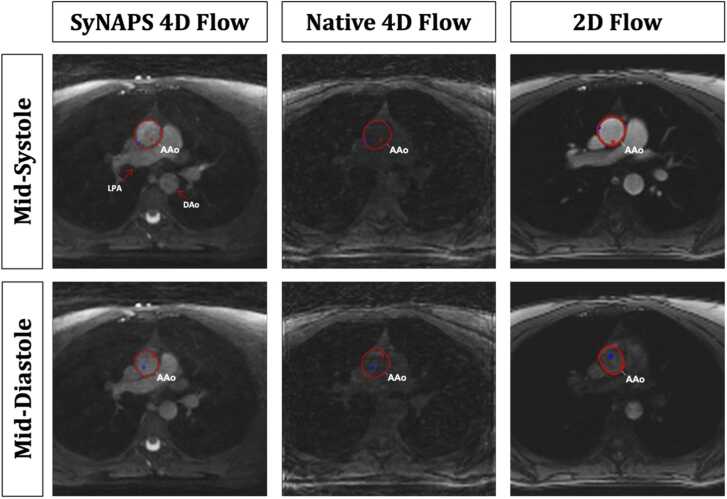
Image contrast between mid-systolic and mid-diastolic phases of the cardiac cycle in the magnitude images for SyNAPS 4D flow, Native 4D flow, and 2D flow datasets of a healthy 26 year old female subject. Image contrast varied in 2D flow, due to inflow effects, while on SyNAPS 4D flow images it remains constant. Native 4D flow provided the worst image contrast of the three techniques. Images and vessel segmentations were captured from the image processing software, and blue and red dots inside the vessel segmentation represent maximum and minimum velocity voxels. 2D: two-dimensional, 4D: four-dimensional, AAo: base of the ascending aorta; DAo: mid descending aorta; LPA: left pulmonary artery, SyNAPS: Synchronization of Neighboring Acquisitions by Physiological Signals.

When looking at the magnitude images over time, the signal intensity of the vessels in SyNAPS 4D flow was consistent throughout the cardiac cycle, while 2D flow magnitude images, due to the in-flow effects, showed clear fluctuations in signal intensity, that resulted in reduced vessel visibility (Fig. 3). Moreover, a consistently low blood signal was observed on the Native 4D flow magnitude images. Supplementary Video S1 shows this effect across the cardiac cycle, for two healthy subjects.

Supplementary material related to this article can be found online at 10.1016/j.jocmr.2024.101006.

The following is the Supplementary material related to this article Video S1.

Video S1 Dynamic visualization of signal intensity in magnitude images from SyNAPS 4D flow, Native 4D flow, and 2D flow datasets. Cardiac motion has been synchronized for all datasets, and the slices used to segment a portion of the AAo and DAo are shown for two subjects. Image contrast varies on 2D flow images, due to inflow effects, while on SyNAPS 4D flow images it remains mostly constant. Native 4D flow provided the worst image contrast of the three techniques..

Image quality analysis of all datasets showed that SyNAPS 4D flow datasets provided an improved vessel delineation compared to Native 4D flow datasets. The image quality scores of SyNAPS 4D flow data provided by the first expert (M.P.) were 1.8 ± 0.4 (AAo) and 1.9 ± 0.3 (DAo). Compared with these scores, the scores from Native 4D flow datasets were had no significant differences (1.5 ± 0.5 for AAo; 1.6 ± 0.7 for DAo, p = 0.03), while 2D flow image quality scores were significantly higher (2.9 ± 0.3 for AAo; 2.7 ± 0.5 for DAo, p < 0.01). Alternatively, the second expert (E.T.) scored SyNAPS 4D flow data (2.4 ± 0.5 for AAo, 2.5 ± 0.5 for DAo) with significantly better vessel delineation when compared to Native 4D flow data (0.7 ± 0.5 for AAo; 1.0 ± 0.0 for DAo, p < 0.01), albeit the image quality scores of SyNAPS 4D flow was still significantly lower than the scores from 2D flow (3.5 ± 0.7 for AAo; 3.4 ± 0.08 for DAo, p < 0.01). Overall, the anatomical information provided by SyNAPS 4D flow was relevant for scoring the quality in vessel delineation in 23 out of 26 datasets for M.P. and for 26 out of 26 datasets for E.T., similarly to the 2D flow datasets (26/26 for M.P., 26/26 for E.T.), and in opposition to Native 4D flow cases (0/26 for M.P., 6/26 for E.T.).

**Fig. 4:**
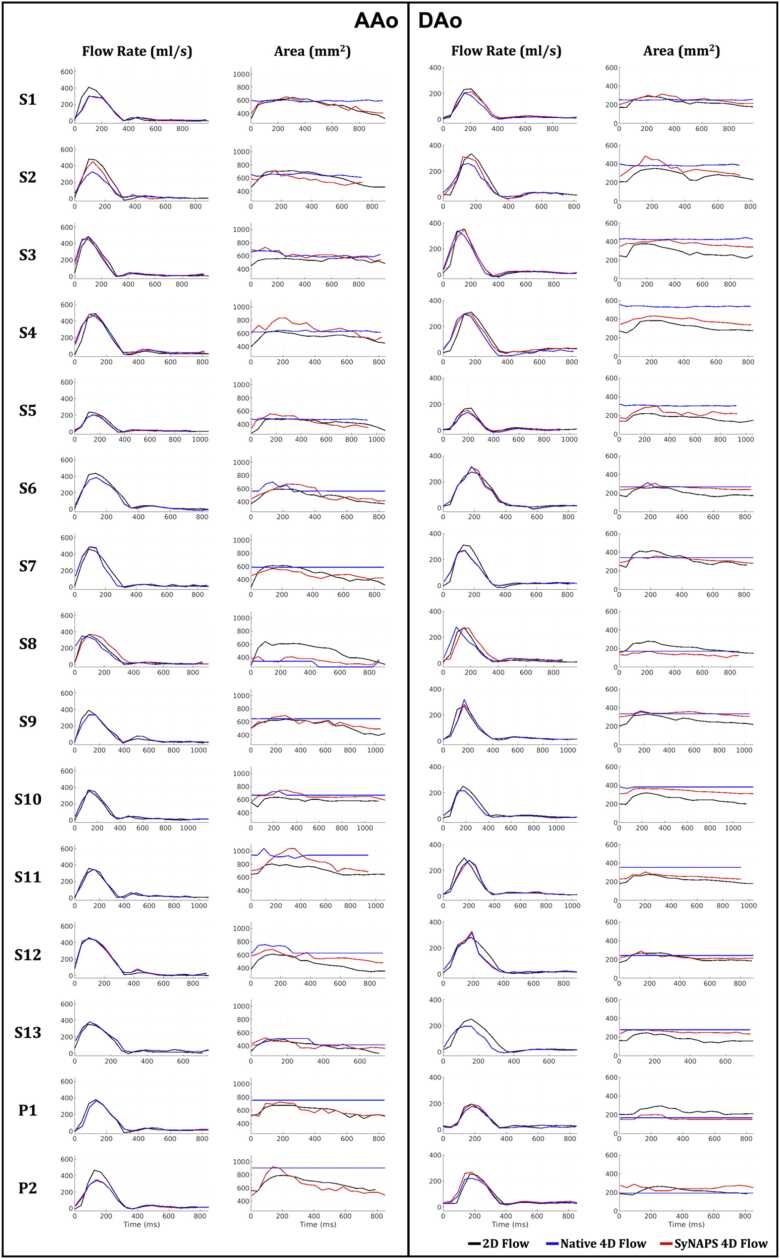
Visualization of flow rate and vessel area across the cardiac cycle for 2D flow, Native 4D flow, and SyNAPS 4D flow. For healthy volunteers S1 to S13 and patients P1 and P2, two vessel locations were chosen, one at the base of the ascending aorta (AAo), and another one in the mid descending aorta (DAo). Black: 2D flow; Blue: Native 4D flow; Red: SyNAPS 4D flow. 2D: two-dimensional, 4D: four-dimensional, SyNAPS: Synchronization of Neighboring Acquisitions by Physiological Signals.

Following segmentation, dynamic changes in vessel area were observed and measured in the SyNAPS 4D flow, as well as for the reference 2D flow datasets, while for Native 4D flow the vessel area appeared static over time (Fig. 4). The error in vessel area between SyNAPS 4D flow and 2D flow was 72.4 ± 37.6 mm^2^ for AAo (14.1 ± 7.1%, p = 0.08) and 57.9 ± 21.1 mm^2^ for DAo (26.0 ± 10.0%, p < 0.01) across all volunteers. Alternatively, when comparing measurements from Native 4D flow with 2D flow, the error in vessel area was 124.6 ± 61.2 mm^2^ for AAo (25.1 ± 10.0%, p = 0.03) and 90.6 ± 52.6 mm^2^ for DAo (40.3 ± 22.4%, p < 0.01). The Dice similarity coefficient between SyNAPS 4D flow and 2D flow vessel segmentations was 0.82 ± 0.17 for the AAo and 0.82 ± 0.15 for the DAo, while the Dice similarity coefficient between vessel segmentations of Native 4D flow and 2D flow was 0.79 ± 0.14 for the AAo and 0.80 ± 0.13 for the DAo. Differences between the SyNAPS 4D flow vs. 2D flow and Native 4D flow vs. 2D flow Dice scores were statistically significant (p < 0.01). The Pearson correlation coefficient between the dynamic vessel area measurements from SyNAPS 4D flow and 2D flow was 0.70 ± 0.22 for the AAo and 0.72 ± 0.44 for the DAo, and the same correlation values obtained from measurements of Native 4D flow and 2D flow were 0.10 ± 0.44 for the AAo and 0.07 ± 0.35 for the DAo.

**Fig. 5:**
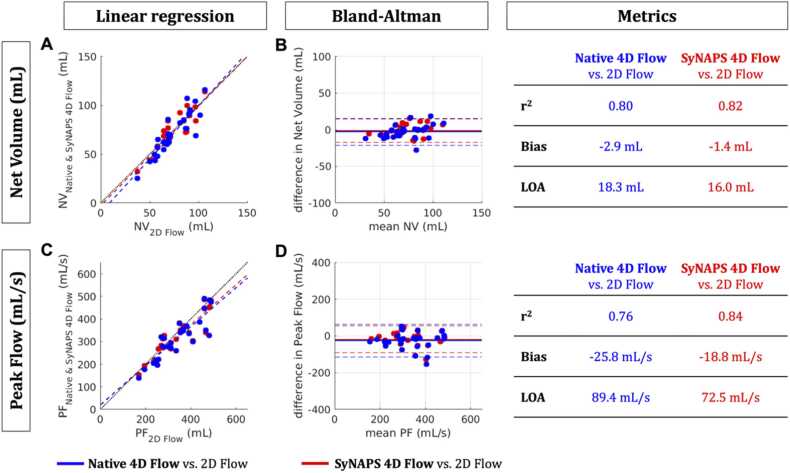
Comparison of net flow **(A-B)** and peak flow **(C-D)** measurements between Native 4D flow vs. 2D flow as well as between SyNAPS 4D flow vs. 2D flow. Linear regression **(A,C)** and Bland-Altman **(B,D)** plots show smaller biases when using SyNAPS 4D flow. **A,C.** Black dotted lines represent identity and dashed colored lines represent the linear regression outcome for each pair. **B,D.** Bias is depicted by solid lines; limits of agreement (LOA) are represented by dashed lines. Blue: Native 4D flow vs. 2D flow; Red: SyNAPS 4D flow vs. 2D flow. r^2^: coefficient of determination. 2D: two-dimensional, 4D: four-dimensional, NV: net volume, PF: peak flow, SyNAPS: Synchronization of Neighboring Acquisitions by Physiological Signals.

Across all analyzed vessels, there was some agreement when comparing flow curves derived from SyNAPS 4D flow, Native 4D flow, and 2D flow datasets (e.g., the AAo in subject S4, Fig. 4), while in some cases both SyNAPS 4D flow and Native 4D flow datasets provided underestimations of the flow rate at peak systole relative to the same measurements from 2D flow datasets (e.g., the DAo in S8). However, in several subjects, there was a better agreement between flow rates measured from SyNAPS 4D flow and 2D flow datasets than those measured from Native 4D flow and 2D flow datasets (e.g., S3 in the AAo). Fig. 4 displays the flow curves and vessel area curves across the cardiac cycle for all subjects included in the study. Linear regressions of the net volume and peak flow rate showed similarly significant correlation between all flow datasets (p < 0.01, Fig. 5). Moreover, Bland-Altman analysis suggested a non-significant reduction of bias and limits of agreement between SyNAPS 4D flow and 2D flow relative to Native 4D flow vs 2D flow (p = 0.7 for net volume and p = 0.4 for peak flow).

## 4 Discussion

In this work, SyNAPS was introduced for the synchronization and reconstruction of consecutively acquired free-running radial whole-heart MRI datasets. A free-running anatomical (FISS) and a flow (PC-MRI) sequence, acquired without contrast agent injection, were combined to reconstruct SyNAPS 4D flow images that provided a better visualization of the cardiovascular anatomy, with improved dynamic segmentation of blood vessels when compared to their equivalent Native 4D flow images.

In SyNAPS 4D flow datasets, the velocity information is derived from the free-running flow sequence, just like in Native 4D flow datasets, while the magnitude information is obtained from 4D FISS datasets, thus providing improved image contrast. Therefore, the main difference between SyNAPS 4D flow and Native 4D flow is the improved anatomical visualization of cardiac structures. SyNAPS 4D flow empowers dynamic segmentation of the vessels of interest and consequently improves flow quantification in these vessels. This is not easily achieved solely with the conventional Native 4D flow technique, as the magnitude information provides very low blood-to-myocardium contrast when performing image acquisition without contrast agent injection. When comparing SyNAPS 4D flow to Native 4D flow imaging, the two blinded reviewers observed an improvement in image quality for vessel delineation when synchronizing sequences with SyNAPS and considered that the magnitude information in SyNAPS 4D flow provided added value to the vessel segmentation. Moreover, the magnitude images obtained from free-running FISS data brought the vessel delineation score from SyNAPS 4D flow dataset closer to the score of the gold-standard 2D flow. Nevertheless, the differences in vessel delineation quality score were significant between SyNAPS 4D flow and 2D flow, suggesting that there is still room for improvement in the choice of anatomical sequence to use in future work. This further strengthens the potential of SyNAPS, as the properties of PT and of this technique may enable the combination of different sequences than the ones presented in this proof-of-concept study.

Compared to the previous studies that leveraged a separately acquired 3D anatomical scan to improve vessel and ventricular segmentation [6], [7], SyNAPS 4D flow does not require contrast agents to delineate anatomical structures. Moreover, the use of PT enabled the synchronization of acquisitions without the need for averaging or interpolating cardiac phases. Additionally, the relationship between the raw PT signals and the underlying physiological motion is expected to be relatively constant across the two sequences unlike traditional self-gating signals which are derived from the image data and therefore are subject to changes based on the contrast of the underlying sequences. This is a unique feature of SyNAPS that could be particularly enabling for synchronizing multiple imaging sequences together to increase information sharing, thus empowering a more comprehensive cardiac MRI exam.

SyNAPS provides a novel solution for cardiac and respiratory synchronization in cardiac MRI that could enable a more comprehensive cardiac magnetic resonance evaluation, where multiple free-running and volumetric sequences are concatenated. These promising initial results motivate further validation of the framework, especially in more complex contexts of heart-rate variability, bulk motion, and respiratory drift.

Using SyNAPS, a contrast-free whole-heart 4D flow acquisition with good blood-to-myocardium contrast that enables the dynamic segmentation of the vessels for improved quantification of blood flow can now be acquired within a fixed scan time. The setup is easy-to-use, does not require respiratory navigators, which reduce scanning efficiency, and shows potential as an alternative to conventional 4D flow techniques when targeting increased image quality without contrast agent injection. Furthermore, the SyNAPS concept could easily be extended to other branches of the free-running framework that target the heart as well as other moving organs, such as the lungs and liver. Other potential applications include T1 [22], T2 [27], and fat fraction mapping [28], with the aim of creating a highly comprehensive MRI-based tool for a synchronized and co-registered assessment of cardiac structure, function, flow, and parametric tissue properties, for an improved and comprehensive clinical assessment of heart disease.

### 4.1 Limitations

The scan time for SyNAPS 4D flow imaging is inherently longer (12:05 min) when compared to the Native 4D flow technique (8:59 min), due to the addition of the free-running FISS sequence (3:06 min). Nevertheless, the addition of the FISS sequence provides excellent delineation of the cardiac anatomy that would otherwise not be obtained using only Native 4D flow. Moreover, the FISS data could be further exploited to assess cardiac structures, such as the coronary arteries [18] and measure function [29]. Furthermore, the scan time presented in this study is also the result of using previously published protocols for both free-running flow and FISS acquisitions. Using optimized adaptations of these sequences, for instance by removing the currently unused SI projections from the acquisition trajectory, would already reduce the scan time.

The dynamic vessel segmentation achieved in SyNAPS 4D flow datasets showed a good correlation with the reference 2D flow dynamic vessel area measurements, suggesting that the anatomical component of SyNAPS 4D flow (from free-running FISS data) was well synchronized with its flow component (from free-running PC-MRI data). However, more rigorous in vitro experiments are needed to address the possible unpredictability of physiological motion patterns (such as bulk motion and respiratory drift) and may help to elucidate further strengths and weaknesses of the SyNAPS technique. Moreover, future work should target the further validation of SyNAPS in patient cohorts, not only to account for more complex pathological anatomies but also to enable a comparison with 4D flow data acquired with gadolinium to investigate the differences with our proposed native contrast approach.

Another potential limitation of this study is the use of rigid fNAV to correct respiratory motion. Here, fNAV was used to correct translational bulk motion, and, as a result, does not account for rotational motion, erroneous motion correction of bright static tissue (such as fat), or the more general non-rigid behavior of respiratory motion, which may introduce artifacts. This may lead to small motion correction errors in the final image datasets. Further developments may therefore aim at extending fNAV to non-rigid motion correction in SyNAPS data or exploring respiratory motion-resolved reconstructions. However, it should be noted that fNAV is not an essential component of the SyNAPS framework, and synchronized datasets can instead be reconstructed in different ways, such as into several respiratory phases to create synchronized five-dimensional imaging volumes [15], [19].

The use of free-running FISS as the anatomical dataset to integrate into SyNAPS 4D flow was chosen because it provides balanced steady-state free precision (bSSFP)-like blood-to-myocardium image contrast [16], [17], [18], [29], but with inherent fat suppression which decreases streaking artifacts in the resulting anatomical image. Beyond demonstrating improved vessel segmentation for quantification of flow measurements, this study did not address the untapped potential of using the free-running FISS for extracting additional anatomical and functional measurements, such as ejection fraction, as this was beyond the scope of the work. Nevertheless, future work should aim at studying the anatomical value that this sequence adds to the final SyNAPS 4D flow dataset and compare it with gold standard sequences. Moreover, FISS suppresses fat for a certain range of repetition time values, which poses a limit on the choice of spatial resolution at 1.5T [18]. However, for higher spatial resolutions, alternative free-running anatomical sequences, such as balanced steady-state free precession with fat saturation pre-pulses [15], [30] or a balanced steady-state free precession sequence incorporating lipid insensitive binomial off-resonant radiofrequency excitation (LIBRE) pulses [31], [32], could be easily integrated into this anatomy and flow framework using SyNAPS.

## 5 Conclusion

This work introduces SyNAPS, a framework that builds toward whole-heart Flow MRI without the need for contrast agents, by synchronizing free-running acquisitions using PT Navigation. We demonstrated the initial feasibility and utility of SyNAPS by combining anatomical and flow MRI sequences that do not require ECG gating or respiratory navigators. We show that the high-contrast anatomical imaging sequence can be leveraged to improve dynamic vessel segmentation, which in turn improves the agreement between reference 2D flow images and 4D flow measurements without the need for contrast agents.

## Funding

M.S. is the PI on Swiss National Science Foundation grants 173129 and 201292 that funded part of this research. C.W.R. is the PI on Swiss National Science Foundation grant PZ00P3_202140 that funded part of this research. J.B. is the PI on the Swiss National Science Foundation grants PCEFP2_194296 and PZ00P3_167871 that funded part of this research. M.M. is the PI on grant T32EB025766 from the National Institute of Biomedical Imaging and Bioengineering of the National Institutes of Health, that funded part of this research. R.v.H. is the PI on the Swiss National Science Foundation grant 32003B_182615 that funded part of this research.

## Author contributions

**Simone Rumac:** Methodology, Writing – review and editing. **Estelle Tenisch:** Methodology, Writing – review and editing. **Milan Prša:** Methodology, Writing – review and editing. **Giulia M.C. Rossi:** Methodology, Writing – review and editing. **Matthias Stuber:** Funding acquisition, Methodology, Project administration, Writing – review and editing. **Adèle L.C. Mackowiak:** Methodology, Writing – review and editing. **Jessica A.M. Bastiaansen:** Methodology, Writing – review and editing. **Christopher W. Roy:** Conceptualization, Supervision, Writing – review and editing. **Michael Markl:** Methodology, Writing – review and editing. **Peter Speier:** Methodology, Writing – review and editing. **Mariana B.L. Falcão:** Conceptualization, Data curation, Formal analysis, Investigation, Methodology, Validation, Visualization, Writing – original draft. **Ruud B. van Heeswijk:** Methodology, Writing – review and editing. **Tobias Rutz:** Methodology, Writing – review and editing. **Mario Bacher:** Methodology, Writing – review and editing.

## Ethics approval and consent

This study was approved by the Research Ethics Committee of the Canton of Vaud (CER-VD) in Switzerland under number 2021-00697 and 2021-00708. All subjects included in this study have consented to participate and to be included in this study.

## Consent for publication

Not applicable.

## Declaration of competing interest

The authors declare the following financial interests/personal relationships which may be considered as potential competing interests. Peter Speier reports a relationship with Siemens Healthineers that includes employment. Mario Bacher reports a relationship with Siemens Healthineers that includes employment. The other authors declare that they have no known competing financial interests or personal relationships that could have appeared to influence the work reported in this paper.
