# Supplementary figures and images for: Combined free-running four-dimensional anatomical and flow magnetic resonance imaging with native contrast using Synchronization of Neighboring Acquisitions by Physiological Signals
Source: J Cardiovasc Magn Reson. 2024 Feb 2;26(1):101006. doi: 10.1016/j.jocmr.2024.101006 (PMC11211232; doi:10.1016/j.jocmr.2024.101006)

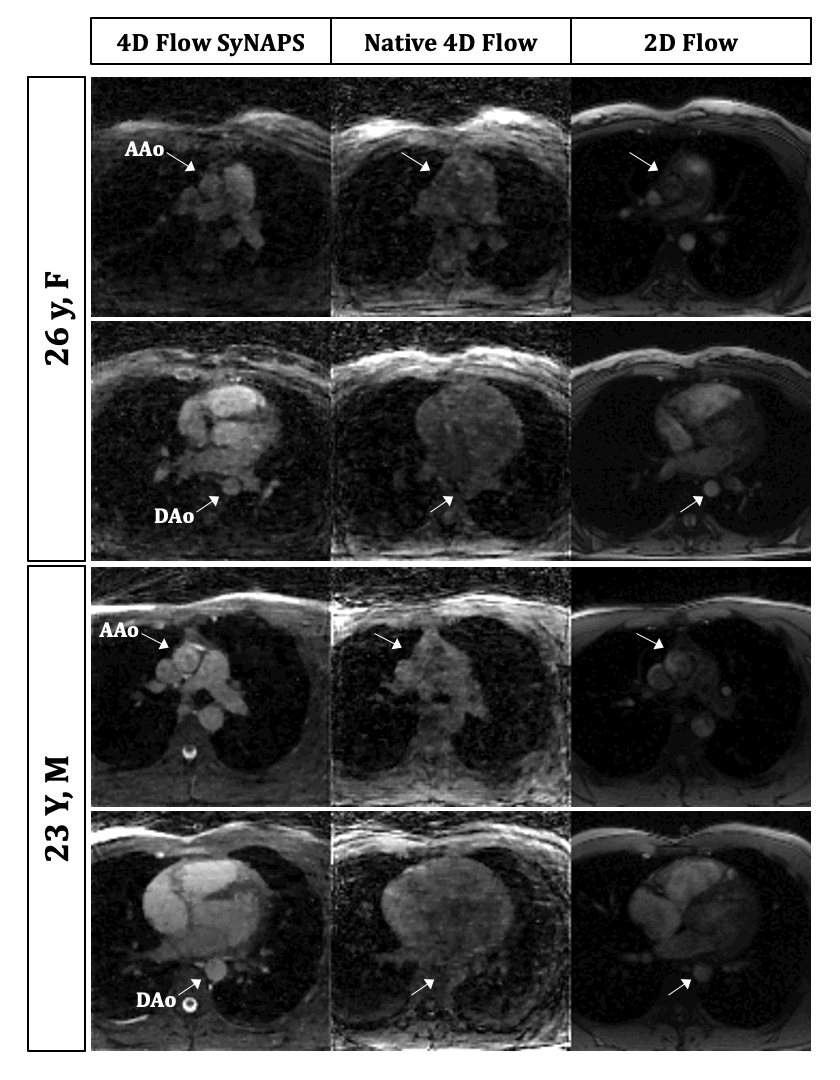

Supplement: Video S1 — Dynamic visualization of signal intensity in magnitude images from SyNAPS 4D flow, Native 4D flow, and 2D flow datasets. Cardiac motion has been synchronized for all datasets, and the slices used to segment a portion of the AAo and DAo are shown for two subjects. Image contrast varies on 2D flow images, due to inflow effects, while on SyNAPS 4D flow images it remains mostly constant. Native 4D flow provided the worst image contrast of the three techniques. [file mmc1.gif]
